# miR-106a Is Downregulated in Peripheral Blood Mononuclear Cells of Chronic Hepatitis B and Associated with Enhanced Levels of Interleukin-8

**DOI:** 10.1155/2015/629862

**Published:** 2015-07-22

**Authors:** Zhongsi Hong, Haiyu Hong, Jian Liu, Xiaobin Zheng, Mingxing Huang, Chunna Li, Jinyu Xia

**Affiliations:** ^1^Department of Infectious Disease, The Fifth Affiliated Hospital of Sun Yat-sen University, Zhuhai, Guangdong 519000, China; ^2^Department of Otolaryngology and Head Neck Surgery, The Fifth Affiliated Hospital of Sun Yat-sen University, Zhuhai, Guangdong 519000, China; ^3^Department of Respiratory Medicine, The Fifth Affiliated Hospital of Sun Yat-sen University, Zhuhai, Guangdong 519000, China

## Abstract

*Aims*. This study aimed to investigate miR-106a expression in peripheral blood mononuclear cells (PBMCs) of chronic hepatitis B (CHB) patients and to analyze the function of miR-106a. *Materials and Methods*. miR-106a expression levels in PBMCs from 40 healthy controls and 56 CHB patients were analyzed by quantitative real-time polymerase chain reaction (qRT-PCR). The luciferase activity assays were used to determine whether miR-106a binds to 3′UTR of IL-8. miR-106a mimics and inhibitors were transfected into healthy PBMCs. IL-8 mRNA and protein levels were detected and determined by qRT-PCR and ELISA, respectively. *Results*. The qRT-PCR results suggested that the PBMC miR-106a levels were decreased in CHB patients. IL-8 was augmented in CHB patients and was inversely correlated with miR-106a levels. The luciferase activity assays indicated that IL-8 is a target of miR-106a. Exogenous expression of miR-106a could significantly repress IL-8 expression at both mRNA and protein levels in PBMCs, whereas miR-106a inhibitor had the opposite effects. *Conclusions*. This study suggested that miR-106a is downregulated in PBMCs of CHB patients and that miR-106a may play an important role in CHB by targeting IL-8.

## 1. Introduction

Hepatitis B virus (HBV) infection is a significant public health problem with approximately 350 million infections worldwide [1]. HBV causes acute and chronic hepatitis and is one of the major causes of cirrhosis and hepatocellular carcinoma (HCC) [2, 3]. HBV is not directly cytopathogenic for infected hepatocytes and the pathogenesis of liver diseases results from complicated interactions between the HBV replication and host immune responses [4, 5]. Abnormal immune responses caused by HBV infection are likely to accelerate liver damage. Thus, exploring the mechanisms of immune responses in chronic hepatitis B (CHB) patients has been the focus of intense research. Proinflammatory cytokines such as TNF-*α*, MCP-1, IL-1a, and IL-6 play a central role in the induction of immune responses in the pathogenesis of various infectious diseases [6–10]. Cytokines released by immune cells in response to virus infections including HBV, respiratory syncytial virus (RSV), and cytomegalovirus (CMV) exert the main functions by recruiting inflammatory cells, constraining virus replication and spread, and inducing adaptive immunity [6–10]. However, when the production of chemokines in the context of viral infection is continuous, it may become harmful to the host. For example, numerous proinflammatory mediators produced and released in association with human RSV challenge, including MCP-1 and IL-6, may promote a viral pathology [8].

Of interest, it has been recently shown that interleukin- (IL-) 8, an important inflammatory cytokine, might be involved in the pathogenesis of HBV infectious diseases, such as CHB. IL-8 is a CXC chemokine able to elicit granulocytes, NK cells, and T cell chemotaxis at the inflammatory site [11]. It is a principal mediator of the inflammatory response to many viruses and bacteria. Clinical studies showed that as the severity of liver inflammation got higher, IL-8 levels increased gradually [12, 13]. This is probably because IL-8 is closely related to natural killer (NK) cells that activate a variety of immune cells to release inflammatory mediators, leading to repeated inflammation and damage of liver function [10]. In addition, IL-8 can reduce HBV sensitivity to IFN-a, thereby counteracting its antiviral action [12].

Previous data had shown that the HBV-encoded regulatory HBX protein is able to transactivate the IL-8 promoter, thereby upregulating its expression [14]. However, recent studies showed that the epigenetic mechanisms are also involved in the regulation of IL-8 gene expression. Epigenetic modifications refer to alterations in gene expression without modification of the DNA sequence, by mechanisms involved with DNA methylation, histone modifications, and miRNA regulation. Variation in methylation status of the IL-8 gene promoters in human cell models investigating periodontitis appears to predispose some subjects to chronic inflammation [15]. Increasing histone acetylation in human intestinal epithelial cells (IEC) was able to potentiate the cellular response to lipopolysaccharide (LPS) as measured by IL-8 protein production [16]. A recent study showed that Aza-TSA (inhibiting DNA methyltransferase and histone diacetyl transferase) treatment significantly alters LPS-induced IL-8 production [17]. miRNAs are small endogenous noncoding RNAs that modulate the expression of genes at the posttranscriptional level by binding to the 3′ untranslated region (3′UTR) of their target mRNAs [18]. IL-8 expression is also regulated by miRNAs. Martinez-Nunez et al. demonstrated that a network of microRNAs (miR-18a, miR-27a, miR-128, and miR-155) modulates IL-8 expression in the asthmatic bronchial epithelium [19]. Furthermore, Dalbeth et al. reported that miR-146a reduced MSU crystal-induced IL-8 gene expression [20].

miRNAs are demonstrated to be involved in the pathogenesis of many diseases, such as viral infection and cancer. miR-199a-5p can facilitate HCV replication by regulating prosurvival pathways [21]. The HCMV-encoded miRNA, hcmv-miR-US25-1-5p, could inhibit viral replication during viral infection [22]. Several miRNAs (e.g., miR-135b, miR-200, and miR-101) were found to be involved in the development of many cancers, such as colorectal cancer, breast cancer, and glioma [23–25]. miRNAs also participate in the development of HBV-related disease. Chen et al. reported that miR-197 is decreased in peripheral blood mononuclear cells (PBMCs) from CHB patients and it is implicated in the pathogenesis of CHB by targeting IL-18, a key regulator in inflammation and immunity [26]. Recently, Zhang et al. examined the miRNA expression profiles in 327 HCC patients, including 327 tumor and 43 adjacent nontumor tissues, and they built a unique 7-miRNA prognosis marker that could significantly predict overall survival (OS) of HCC patients [27]. miR-485-5p represses HCC invasive and metastatic capacities by targeting EMMPRIN expression [28]. miR-582-5p was demonstrated to inhibit proliferation of hepatocellular carcinoma by targeting CDK1 and AKT3 [29].

During viral infection, virus associated molecular patterns were recognized by Toll-like receptors (TLRs), followed by recruitment of their distinct adaptor proteins, sequentially activating signalling cascades to induce cytokine production [30]. miRNAs participate in the process of HBV infection mainly by regulating TLRs and cytokine signaling pathway [31, 32]. Repression of the let-7 family relieves IL-6 and IL-10 mRNAs from negative posttranscriptional control in the TLR4 signaling pathway [33]. miR146a impairs the IFN-induced anti-HBV immune response by downregulating STAT1 in hepatocytes [34]. Thus, it is important to investigate the role of miRNAs in immune response during HBV infection.

Our group took advantage of miRNA expression profile to identify differentially expressed miRNAs in PBMCs from CHB patients and healthy controls (data not shown), among which miR-106a was found to be significantly downregulated. In the present study, we verified the expression of miR-106a by real-time PCR in CHB patients and investigated its possible role. Our results provide a new concept to understand the role of miR-106a in immune responses induced by HBV infection.

## 2. Materials and Methods

### 2.1. Subjects

Blood samples were collected from 56 patients with CHB with abnormal liver function as well as 40 healthy people. Patients with CHB were selected from the clinic or hospitalization unit at the Department of Infection of the Fifth Affiliated Hospital of Sun Yat-sen University during January 2012 and May 2015. The standards of diagnosis complied with the standards for diagnoses of CHB prevention and treatment guidelines recommended by liver disease of the Chinese medical association. All patients with a history and clinical features of drug-induced liver injury, alcoholic hepatitis, and steatohepatitis as well as those treated with nucleotide or nucleotide-analog antiviral or immunomodulatory drugs were excluded from this study. Patients had no history of treatment for HBV prior to the study. This study was approved by the human ethics committee of Sun Yat-sen University. Informed consent was obtained from all subjects studied.

### 2.2. Determination of Alanine Aminotransferase Activities

The activities of ALT were calculated spectrophotometrically in serum, using Beckman Coulter kits by autoanalyzer (Unicel D × C 800 Synchron, Brea, California, USA). The results were expressed as units per liter (Unit/L).

### 2.3. qRT-PCR for Detecting miRNA and mRNA Expression

PBMCs were isolated from heparinized blood by density gradient centrifugation with a lymphocyte separation medium. Total PBMCs RNA was isolated using the Trizol reagents as instructed (Invitrogen, San Diego, CA, USA), and RNA concentrations were determined with a NanoDrop instrument (NanoDrop Technologies, Wilmington, DE). RNA quality and quantity were monitored by ethidium bromide staining and by UV absorbance.

The expression of miR-106a was determined by miScript SYBR Green PCR kit (Qiagen, Germany). The miRNA amplification was conducted on a Light Cycler (Roche Diagnostics). The 20 *μ*L PCR reactions consisted of 10 *μ*L of 2 × QuantiTect SYBR Green PCR Master Mix, 2 *μ*L of 10 × miScript Universal Primer, 2 *μ*L of 10×miScript Primer Assay, 1 *μ*L of RNA, and 2 *μ*L of RNase-free water. Mammalian U6B small nuclear RNA in the PBMCs was used to normalize the miRNA expression level. The miR-106a and U6B primers were purchased from Qiagen.

For quantification of IL-8 mRNA, 1 *μ*g of extracted RNA for each sample was treated with RQ1 RNase-free DNase (Promega, Madison, WI) for 1 h at 37°C and used as a template for cDNA synthesis using Reverse-Transcribe Kit (Promega Co., Madison, WI, USA) in accordance with the manufacturer's protocol. The relative IL-8 expression level was determined by using the following specific primers 5′- ACTCCAAACCTTTCCACC -3′ and 5′- AACTTCTCCACAACCCTC -3′ [35] and normalized to glyceraldehyde phosphate dehydrogenase (GAPDH). The PCR amplification was performed with a volume of 20 *μ*L containing 10 *μ*L SYBR qPCR Mix (Takara, Dalian, China). The relative expression of miR-106a and IL-8 (defined as fold change) was calculated by the 2^−ΔΔCt^ (ΔCt = Ct^miR-106a/IL-8^ − Ct^U6B/GAPDH^; ΔΔCt = ΔCt^sample^ − ΔCt^conotrol^). The real-time PCR experiments were repeated for three times.

### 2.4. Cell Culture and Transfection

The isolated PBMCs were cultured in RPMI 1640 medium supplemented with 10% fetal calf serum (FCS) and penicillin/streptomycin in U-bottom 24-well plates at 37°C with 5% carbon dioxide. The human embryonic kidney (HEK) 293 cells were grown in DMEM medium supplemented with 10% FCS. The hsa-miR-106a mimics, hsa-miR-106a inhibitor, and unrelated sequence positive control (control mimics) and negative control (control inhibitors) were purchased from GeneCopoeia (Jiangsu, China). PBMCs were transfected using Lipofectamine 2000 reagents according to the manufacturer's instructions (Invitrogen). Then, the cells were subjected to analysis of miR-106a and IL-8 mRNA expression as above. And the protein expression levels of IL-8 were measured in supernatant by ELISA (R&D Systems, Minneapolis, MN, USA).

### 2.5. Luciferase Assay

To generate luciferase reporter constructs, the 3′-UTR regions of IL-8 containing the predicted target sites of miR-106a were cloned into pMIR-REPORT miRNA expression reporter vector (Yinrun, China) by using synthesized fragments (5′- CTAGTTGTTGTGAGGACATGTGGAAGCACTTTAAGTTTTTTCATCATAA -3′ and 5′- AGCTTTATGATGAAAAAACTTAAAGTGCTTCCACATGTCCTCACAACAA -3′). The corresponding mutant plasmid was also constructed with the fragments (5′- CTAGTTGTTGTGAGGACATGTGGAACCCGCAAAAGTTTTTTCATCATA A -3′ and 5′- AGCTTTATGATGAAAAAACTTTTGCGGGTTCCACATGTCCTCACAACAA -3′). For luciferase reporter assays, pMIR-IL-8-3′-UTR (WT or MUT) plasmid was cotransfected with miR-106a mimics into the HEK293 cells as previously described [36, 37]. The pMIR-REPORT-*β*-gal control vector was also transfected as an internal control. Then, the cells were collected and determined by using a Dual-Luciferase Reporter Assay System (Promega).

### 2.6. Statistical Analysis

All statistical analyses were conducted with the Statistical Product and Service Solutions (SPSS) version 16.0 (SPSS, Chicago, IL, USA). The real-time PCR results were analysed using the 2^−ΔΔCt^ method. The differences of miR-106a and IL-8 expressions were assessed by using unpaired Student'*t* test. For determination of correlation between different variables Spearman's correlation coefficient was used. *p* value less than 0.05 was considered to be statistically significant.

## 3. Results

### 3.1. The Expression of miR-106a in PBMCs Is Downregulated from CHB Patients and Is Negatively Correlated with Serum Alanine Aminotransferase (ALT) Level

**Figure 1:**
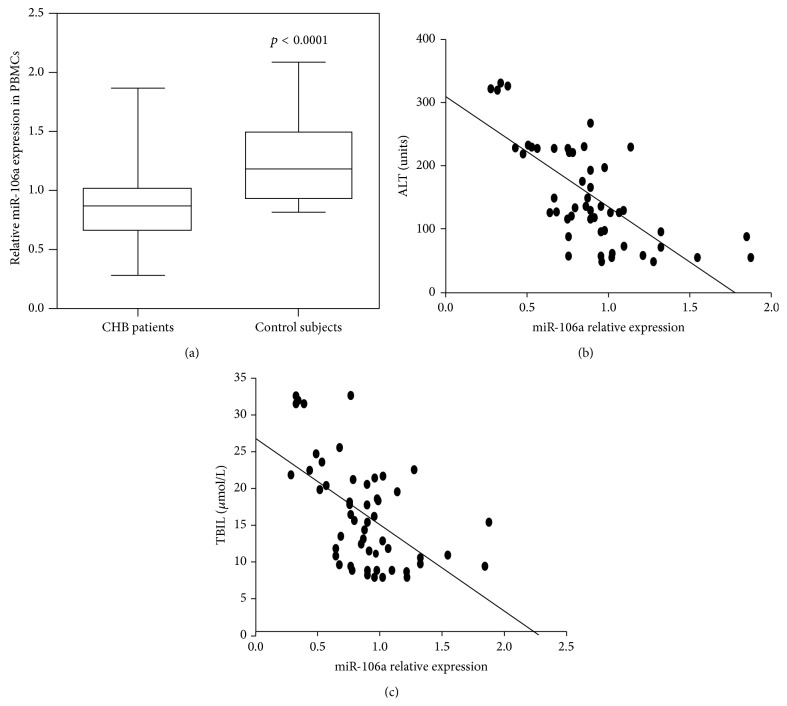
miR-106a is downregulated in PBMCs of CHB patients and is negatively correlated with serum alanine aminotransferase (ALT) level. (a) The expression levels of miR-106a in PBMCs of 56 CHB patients and 40 healthy controls were determined by qRT-PCR. The expression levels were normalized to U6B. (b) PBMC miR-106a was negatively correlated with serum ALT levels. (c) PBMC miR-106a was negatively correlated with TBIL levels.

To identify the miRNAs potentially associated with HBV infection, the expression levels of miR-106a from 56 CHB patients and 40 healthy controls were determined by qRT-PCR. The clinical data of 56 CHB patients are shown in Table 1. We observed that the miR-106a expression level was decreased in PBMCs from CHB patients compared with that in healthy controls (Figure 1(a)). Serum alanine aminotransferase (ALT) and TBIL have been widely recognized as an important marker of liver disease. Here, we analyzed the relationship between miR-106a expression, serum ALT, and TBIL level in CHB patients. The results showed that miR-106a expression was inversely proportional to serum ALT level (*r* = −0.693, *p* = 0.000) and TBIL level (*r* = −0.545, *p* = 0.000) (Figures 1(b) and 1(c)).

### 3.2. Serum IL-8 Protein and mRNA Were Augmented in CHB Patients and Were Positively Correlated with Serum ALT

**Figure 2:**
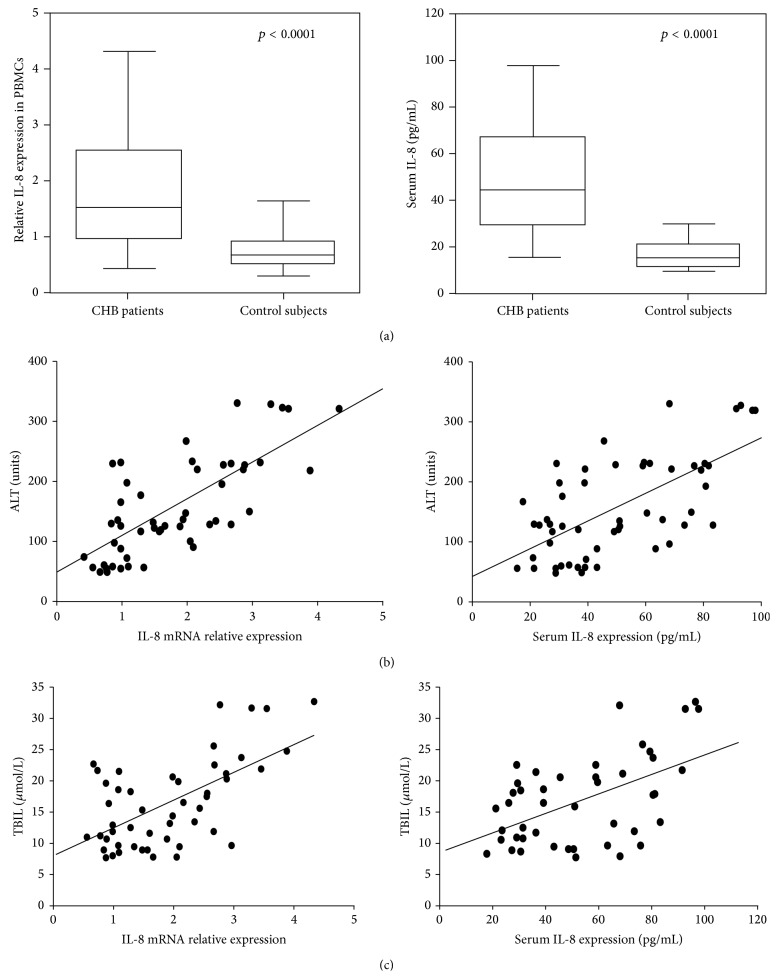
Serum IL-8 protein and mRNA levels were significantly increased in CHB patients and were positively correlated with serum ALT. (a) Serum IL-8 and PBMCs IL-8 mRNA of 56 CHB patients and 40 healthy controls were assessed by ELISA and qRT-PCR. (b) Both serum IL-8 and PBMCs IL-8 mRNA were positively correlated with serum ALT levels. (c) Both serum IL-8 and PBMCs IL-8 mRNA were positively correlated with TBIL levels.

To evaluate whether IL-8 protein and mRNA were increased in CHB, the levels of IL-8 protein and mRNA in CHB patients and healthy individuals were analyzed. As expected, IL-8 protein and mRNA were significantly higher in CHB patients than that of health controls (Figure 2(a)). We also investigated whether IL-8 expression was related with serum ALT in CHB patients. And the analysis results revealed that there was positive correlation between serum IL-8 and ALT levels (*r* = 0.569, *p* = 0.000) (Figure 2(b)). IL-8 mRNA in PBMCs was also positively correlated with serum ALT level (*r* = 0.645, *p* = 0.000) (Figure 2(b)). In addition, serum TBIL levels were found to be significantly correlated with IL-8 mRNA level (*r* = 0.498, *p* = 0.000) and serum IL-8 protein level (*r* = 0.476, *p* = 0.000) (Figure 2(c)).

### 3.3. IL-8 Is One Direct Target of miR-106a

**Figure 3:**
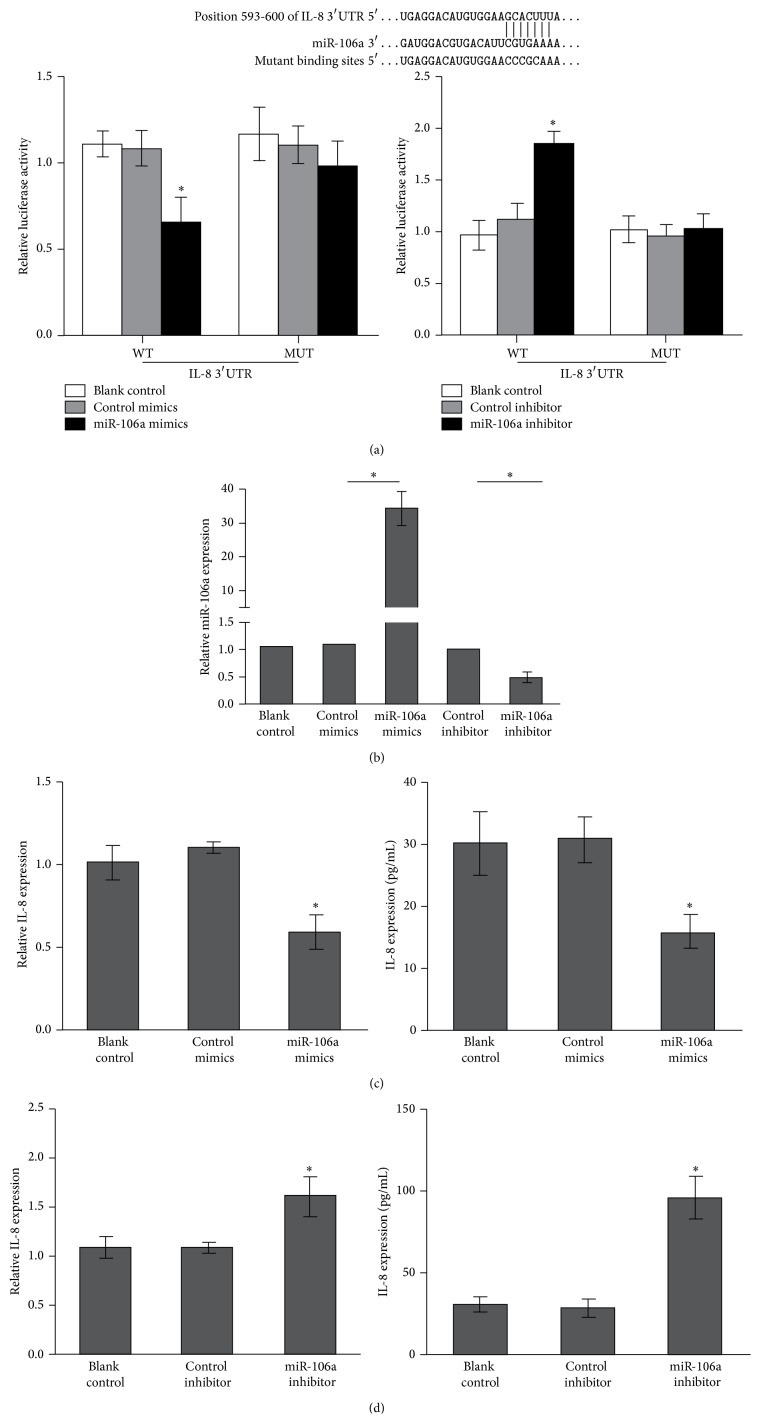
IL-8 is a direct target of miR-106a. (a) Up: potential sites in IL-8 3′UTR targeted by miR-106a. 3′UTR of IL-8 was cloned into a luciferase reporter vector. Mutated sequences were generated in the seed regions to abolish binding of the corresponding miRNAs. Down: HEK293 cells were cotransfected with miR-106a mimics or inhibitor and the luciferase reporter constructs harboring IL-8 or mutant IL-8 3′UTR fragments. The luciferase reporter assays were performed 48 h after transfection. The luciferase activities were measured and normalized to a Renilla luciferase activity. (b) miR-106a expression level was determined after transfection with miR-106a mimics or inhibitor. (c) IL-8 mRNA and protein levels after transfection with miR-106a mimics were assessed by qRT-PCR and ELISA. (d) IL-8 mRNA and protein levels after transfection with miR-106a inhibitor were assessed by qRT-PCR and ELISA. ^*∗*^ *p* < 0.05.

Using the Targetscan 6.0, we found that IL-8 was one of the possible target genes for miR-106a. To demonstrate it, a luciferase assay was employed for detailed analysis. HEK293 cells were cotransfected with the IL-8 3′-UTR construct (WT) or its mutant (MUT) and miR-106a mimics or miR-106a inhibitor, followed by a measurement with the luciferase reporter assay (Figure 3(a)). As expected, miR-106a mimics decreased IL-8 translation, whereas miR-106a inhibitor increased the activity of IL-8 translation (Figure 3(a)). However, the luciferase activity of the IL-8 mutant was not influenced by miR-106a mimics or miR-106a inhibitor (Figure 3(a)). Next, we determined the effect of miR-106a on IL-8 mRNA and protein expression levels by qRT-PCR and ELISA. We firstly determined the efficiency of the transfection of miR-106a mimics and inhibitor. As is shown in Figure 2(b), transfection of miR-106a mimics into PBMCs can significantly increase its expression, while miR-106a inhibitor downregulated its expression level. We next assessed the mRNA and protein expression levels of IL-8 in the cells. IL-8 mRNA and protein levels were significantly downregulated in healthy PBMCs transfected with miR-106a mimics compared with the cells transfected with control mimics, whereas the expressions of IL-8 were significantly upregulated in cells transfected with miR-106a inhibitors compared with cells transfected with control inhibitors (Figures 3(c) and 3(d)).

### 3.4. miR-106a Is Negatively Correlated with IL-8 in PBMCs

**Figure 4:**
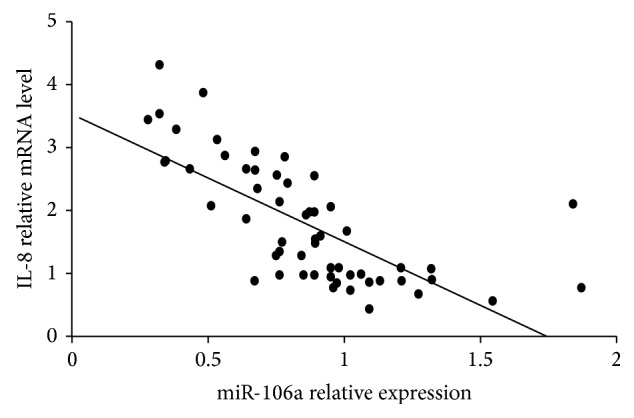
miR-106a had a negative correlation with IL-8 mRNA levels in patients with chronic hepatitis B infection.

Next, we assessed the relationship between miR-106a and IL-8 mRNA levels in PBMCs of CHB patients. Results showed that miR-106a was negatively correlated with IL-8 mRNA levels in PBMCs from CHB patients (*r* = −0.700, *p* = 0.000) (Figure 4).

## 4. Discussion

miRNAs play an important role in HBV infection and HBV-related disease, such as HCC, and changes in miRNA expression patterns are important indicators of these diseases. PBMC miRNAs are also suggested to be potential biomarkers. Results from recent research have shown that the miRNA expressions of PBMC, such as hsa-miRPlus-E1063, hsa-miRPlus-E1236, ebv-miR-BHRF1-2, hsa-miRPlus-A1098, hsa-miR-1267, and hsa-miRPlus-E1163, were associated with the severity of HBV-induced liver disease and therapeutic outcome of IFN-*α* therapy in CHB patients [38]. Xing et al. have demonstrated significantly different expression profiles of miRNA molecules during IA phases of CHB, including hsa-miR-548ah-5p, hsa-miR-4804-3p, hsa-miR-483-3p, hsa-miR-3607-3p, and hsa-miR-44475. miR-548ah- 5p can influence the molecular function of IFN-*γ* through targeting IFN-*γ*R1 and inhibiting its expression [39]. Our group also used miRNA expression profile to identify differentially expressed miRNAs in PBMCs from CHB patients and healthy controls (data not shown). The microarray results showed that miR-106a was significantly downregulated in CHB patients. In the current study, we further verified the expression of miR-106a in PBMCs of CHB patients by real-time PCR assays and demonstrated that miR-106a is downregulated in CHB patients compared with that of healthy controls. Furthermore, clinical analysis revealed that it is negatively correlated with serum ALT and TBIL in CHB patients. Thus, our results firstly revealed that the abnormal expression of miR-106a in PBMCs may have an important role in the damage of liver function during chronic HBV infection.

HBV is a typically noncytopathic virus that does not directly damage infected cells. The pathogenesis is largely mediated by immune responses following HBV infection [4, 5]. IL-8, a member of the cysteine-x-cysteine (CXC) chemokine subfamily, is an important mediator of inflammatory responses to many viruses and bacteria, as it recruits granulocytes, NK cells, and T cells at the inflammatory sites [40, 41]. Recent evidence has suggested that this chemokine may be involved in immune pathogenesis of HBV infection and in the resistance of HBV to IFN-*α*. Moreover, it is strictly correlated with malignant features of tumors, a frequent event in chronic HBV. By using computational methods, we predicted that miR-106a is highly likely to interact with the 3′UTR of IL-8. To confirm whether IL-8 is a direct target of miR-106a, we subcloned IL-8 3′UTR containing the target sequences into a luciferase reporter vector, and the corresponding pMIR-3′-UTR-MUT was also constructed. The vectors were then cotransfected into the HEK293 cells with miR-106a mimics or inhibitor. It was found that miR-106a binds to 3′UTR of IL-8. Next, we transfected mimics and inhibitors of miR-106a into healthy PBMCs. Our results showed that exogenous expression of miR-106a could significantly repress IL-8 expression at both the mRNA and protein levels, whereas miR-106a inhibitor had the opposite effects. Further statistical analysis showed that miR-106a is negatively related to IL-8 expression in PBMCs from CHB patients. These data revealed that miR-106a may be an effective strategy for regulating the expression of IL-8.

In summary, our study firstly provides the evidence that miR-106a is downregulated in PBMCs of CHB patients, and miR-106a expression levels were negatively associated with the severity of HBV-induced liver disease. In addition, our data demonstrated that miR-106a downregulates IL-8 expression by targeting its 3′UTR. We believe that our study may help to develop a better understanding of clinical characterization and pathogenesis of CHB.

## Figures and Tables

**Table 1:** Characteristics of CHB patients and control subjects.

	CHB (56)	HC (40)
Sex (male/female)	40/16	27/13
Ages (year)	32.3 ± 6.6	22.8 ± 6.7
ALT (units/L)	157.5 ± 81.1	22.3 ± 8.0
TBIL (*µ*mol/L)	16.7 ± 7.0	10.5 ± 3.8
HBV DNA (log^10^copies/mL)	5.9 ± 1.6	Undetectable

CHB, chronic hepatitis B; HC, healthy control; ALT, alanine aminotransferase; TBIL, total bilirubin; HBV, hepatitis B virus.
